# Probing the role of *Coniferin* and *Tetrahydrocurcumin* from Traditional Chinese medicine against PSAT1 in early-stage ovarian cancer: An *in silico* study

**DOI:** 10.1371/journal.pone.0313585

**Published:** 2025-02-06

**Authors:** Jia Zhang, Shalesh Gangwar, Nagmi Bano, Shaban Ahmad, Mohammed S. Alqahtani, Khalid Raza

**Affiliations:** 1 Shanxi Province Cancer Hospital/Shanxi Hospital Affiliated to Cancer Hospital, Chinese Academy of Medical Sciences/Cancer Hospital Affiliated to Shanxi Medical University, Taiyuan, China; 2 Department of Computer Science, Computational Intelligence and Bioinformatics Lab, Jamia Millia Islamia, New Delhi, India; 3 Radiological Sciences Department, College of Applied Medical Sciences, King Khalid University, Abha, Saudi Arabia; 4 BioImaging Unit, Space Research Centre, University of Leicester, Leicester, United Kingdom; University of Mashreq, IRAQ

## Abstract

Ovarian cancer, a formidable gynaecological malignancy, poses a significant global health challenge, and it is characterised by late-stage diagnosis and a high mortality rate. Even in its early stages, when treatment choices are scarce, ovarian cancer is still a complicated cancer to treat. In this work, we used computational approaches to find putative Traditional Chinese Medicine (TCM) inhibitors that target Phosphoserine Aminotransferase 1 (PSAT1), a crucial enzyme linked to the development of early-stage ovarian cancer. Using a methodical screening approach, we chose a panel of TCM compounds and prepared them, expected to interact with PSAT1. We next evaluated the binding affinities using molecular docking, which helped to identify Coniferin and Tetrahydrocurcumin compounds as potent inhibitors with the docking score of -8.8 kcal/mol and -8.9 Kcal/mol, respectively, and compared to the native ligand. The inhibitory effects of Coniferin and Tetrahydrocurcumin compounds were evaluated with the Pharmacokinetic studies and compared with the standard values, which resulted in an utter performance against each descriptor of the QikProp and performed the Molecular Interaction Fingerprints that resulted in the most interaction residues with counts were 4GLY, 4ASN, 4HIE, 4SER, 4THR, 3ARG and many more. Further, 100ns MD Simulation was performed in neutralised water, resulting in entirely stable deviations, fluctuations, and many intermolecular interactions, and the MM\GBSA studies on all 1000 trajectories have supported the complex’s stability. The computational studies have shown a completely stable performance that supports Coniferin, and Tetrahydrocurcumin can be a potent inhibitor of PSAT1. However, further experimental works are needed to confirm this study.

## 1. Introduction

Globally, ovarian cancer is a significant health concern due to its high death rate, which is mainly caused by late-stage diagnosis and few effective treatment choices. According to GLOBOCAN’s 2020 estimations, 314,000 women were diagnosed with ovarian cancer in 2020, resulting in 207,000 fatalities [1]. Ovarian cancer is the eighth most common cancer in women, both in terms of incidence and fatality [2]. Notably, industrialised economies in Europe and North America have the greatest ovarian cancer rates, whereas African countries have a lower overall frequency. It is also a leading cause of cancer-related deaths in India due to difficult diagnosis in the early stages. Survival for 5 years when ovarian cancer is diagnosed in stage-I is 94%, constituting only 15% of all diagnoses, whereas 62% of cases of ovarian cancer are diagnosed in stages III and IV, where the 5-year survival is only 28% [3,4]. Current medicines’ efficacy limitations and resistance development highlight the necessity for innovative therapeutic approaches, even with their breakthroughs. Traditional Chinese medicine (TCM) offers a fascinating route for investigating possible inhibitors targeting Phosphoserine Aminotransferase 1 (PSAT1) in early-stage ovarian cancer [5]. TCM’s holistic approach and abundance of natural substances are leveraged in this regard [6]. Recent studies identified PSAT1 as a viable therapeutic biomarker in the early stages of ovarian cancer [7,8].

PSAT1 is essential for the serine biosynthesis pathway, which supports the growth and survival of cancer cells, especially those in the early stages of ovarian cancer. PSAT1, a pyridoxal-phosphate-dependent aminotransferase of class V, is an essential enzyme in the serine-glycine biosynthetic pathway [7]. The second stage converts 3-phosphohydroxypyruvate (3-PHP) to L-phosphoserine via a glutamate-linked transamination process. In PSAT1, Trp107, present in the active site, plays a crucial role in the cofactor orientation and catalysis of 3-PHP [9]. PSAT1’s regulatory involvement in carcinogenesis and malignant progression has been extensively studied. PSAT1 inhibition causes DNA damage and apoptosis, emphasising its importance. Furthermore, PSAT1 plays a role in resistance in melanoma, pancreatic, and non-small cell lung carcinoma [10]. PSAT1 expression is associated with aggressive metastasis and poor clinical outcomes in triple-negative breast cancer [11]. These findings highlight PSAT1’s diverse role in cancer biology, making it a potential target for therapeutic approaches.

The study aims to find a novel approach to early-stage ovarian cancer treatment using computational identification and validation of possible TCM inhibitors targeting PSAT1 [9,12,13]. Through the use of computational techniques such as virtual screening, molecular docking, and simulations, this study seeks to screen a variety of TCM molecules against PSAT1’s active region. The combination of experimental validations and computational methods in drug discovery and development has great potential to accelerate the discovery of new treatments. By providing light on possible TCM-based inhibitors targeting PSAT1, this publication seeks to further the knowledge in the area and open the door for additional preclinical and clinical research in the search for better treatment approaches for early-stage ovarian cancer [14,15].

In this study, we used the TCM library of 238 compounds, docked them with the PSAT1 along with the native ligand, and tried to compare the binding affinity of the native and TCM compounds. Further, we computed the interaction fingerprints of the compounds and pharmacokinetics studies using the QikProp tool. The 100ns MD simulation was also performed to evaluate the deviation fluctuation and Intermolecular interactions.

## 2. Methods

**Fig 1:**
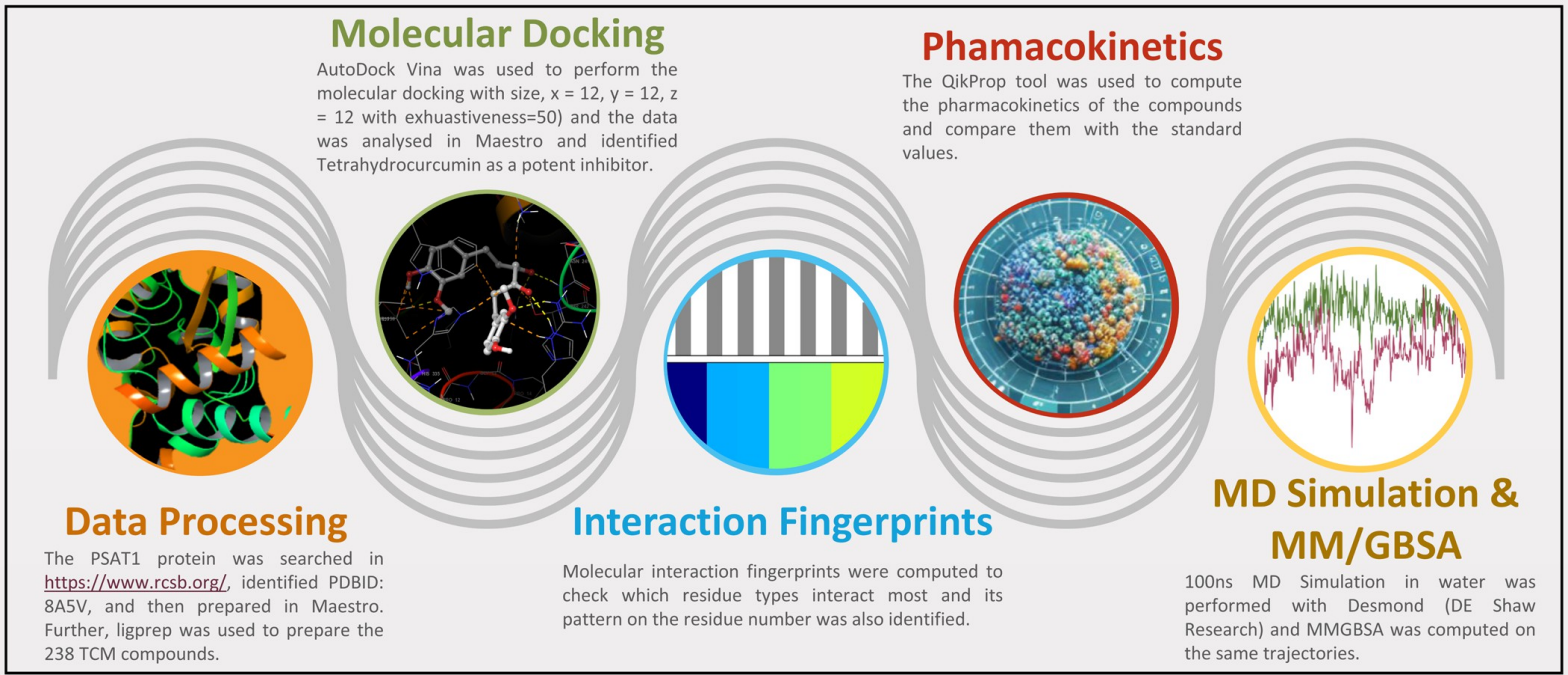
Showing the graphical abstract of the methodology followed in the complete study.

The whole methodology is depicted in Fig 1 to make the study clear. Further, the detailed parameters for the mentioned tasks are as follows-

### 2.1 Protein data collections and preparations

The structure of human phosphoserine aminotransferase (PSAT1) bound with PMP (Pyridoxamine Phosphate) was obtained from RCSB PDB (PDBID: 8A5V) which contains eight protomers arranged as four dimers in the asymmetric unit. The monomers are well-ordered and each monomer adopts the typical α/β fold seen in fold-type I PLP-enzymes, divided into two domains. The larger domain, spanning residues 19–265, features seven β-strands surrounded by six α-helices, playing a key role in forming the dimer interface. The smaller domain, composed of residues 266–307 along with the first 18 N-terminal residues, includes three antiparallel β-strands and three α-helices. These eight monomers are arranged into four stable S-shaped dimers[9].

The crystal structure was imported using the Schrodinger suite with Maestro software 13.4 version [9,16,17]. Subsequently, chains A and B (Domain) were retained, and other heteroatoms were removed. The protein preparation workflow of Schrödinger Suite 2022–4 in Maestro 13.4 was employed with the protein preparation wizard tool, constituting several steps. Firstly, missing side chains were filled in, and bond orders were assigned using the Chemical Component Database (CCD). Hydrogens were replaced, disulfide bonds were created, and missing loops were filled using Prime [18]. Possible states at pH 7.4±2.0 were generated, and H-bond assignments were optimised using PROPKA [19,20]. Finally, the structure was minimised using the OPSL4 forcefield and deleted the water molecules beyond 5Å to heteroatoms [21,22].

### 2.2 Ligand library collection and preparations

A literature survey was conducted to identify potential Traditional Chinese medicine (TCM) inhibitors of PSAT1. TCM used in cancer treatment such as *Allium sativum*[34], *Astragalus membranaceous* (Huang Qi)[35], *Panax ginseng* (Ren Shen)[36], *Curcuma longa*[37] (list of molecules obtained from Dr. Duke’s Phytochemical and Ethnobotanical databases: https://phytochem.nal.usda.gov/), Toad venom (Chan Su) [23], and *Mylabris phalerata* (Ban mao)[38] were considered. 238 molecules were downloaded from PubChem in 2D in SDF format, of which 217 molecules (<600g/mol) were used for further processing. The ligands were prepared using the LigPrep tool of Schrödinger Suite 2022–4 in Maestro 13.4. The force field OPLS4 was applied, and possible states at pH 7.4 ±2 and the heterostate were generated using Epik, and the desalt and generated tautomers were kept [20,21]. The stereoisomers computations kept retaining specified chiralities and generated 10 per original compound, resulting in 417 ligands in SDF format.

### 2.3 Molecular docking

Molecular docking is a computational technique in drug design exploring how molecules interact by predicting the preferred orientation of the small molecules when bound to a receptor to understand potential interactions. Molecular docking was performed using AutoDock Vina v1.1.2 with configuration parameters (center_x = -3.8450, center_y = -55.9037, center_z = 4.6202, size_x = 12, size_y = 12, size_z = 12, exhuastiveness = 50) [24–26]. The grid dimensions were defined around the co-crystallized ligand, which describes the active site targeted for inhibition. Generating the grid box with parameters for docking was set using Biovia Discovery Studio Visualizer v21.1.0.20298. Ligand and protein were converted to .pdbqt using OpenBabel v2.4.0, and protein-ligand interactions after docking was analysed using Schrodinger Maestro(academic version) [17]. AutoDock Vina was used as Glide is not available in the academic version of Maestro.

### 2.4 Pharmacokinetics and molecular interaction fingerprints

Pharmacokinetics is the study of how the body interacts with drugs, which provides information on drug absorption, distribution, metabolism, and excretion and helps optimise drug dosages for efficacy and safety. The pharmacokinetics of the Coniferin and Tetrahydrocurcumin were computed using the QikProp tool in Maestro and compared with the standard values of the QikProp [27]. Molecular interaction fingerprints are a tool for characterising and comparing molecular interactions that capture the patterns of interaction between a ligand and a target protein, aiding in the analysis of binding sites. We used the Interaction Fingerprints tool in Maestro, where we selected the receptor-ligand complex option, counted any interactions, and generated the fingerprints by keeping the bond distances at their default. The fingerprints were exported to the matrix, the main plot was coloured by sequence number, and the non-interacting residues were removed [17].

### 2.5 Molecular dynamics simulation and molecular mechanics, general born surface area (MM\GBSA) studies

The Molecular Dynamics simulation is a facilitated computational analysis for evaluating the complexes, which can be used to understand how biomolecules behave and interact over the period. It also helps to study the functions of biomolecules and predicts the reactivity, stability, and interactive nature with other molecules, which helps in designing new compounds or validating the existing ones and behavioral study of biomolecules in diseases [28]. We have used the academic version of Desmond, developed by DE Shaw Research (https://www.deshawresearch.com/) for MD Simulations [17,29]. The MD simulation is divided into two parts: the first is to build the complete environment, and the second is the production run for MD simulations. The System Builder tool of Maestro is used for building a complete system with an orthorhombic box with an SPC water model in boundary box size of 10 × 10 × 10 Å in buffer, also eliminates the ions and salts within 20 Å, and 1Cl^-^ was added in case of PMP whereas 2Cl^-^ were added in cases of Tetrahydrocurcumin and Coniferin to neutralise the system. The system builder yielded 75687, 75744 and 75740 atoms for the PMP, Coniferin and Tetrahydrocurcumin, respectively. The production run maintaining MD simulation parameters such as a duration of 100 ns and recording intervals of 100 ps that generated 1000 frames per simulation was kept. Further, the NPT ensemble class was kept at an energy level of 1.2 at 300K temperature and 1.01325 bar pressure [30]. The resultant trajectories were analysed post-MD simulations using the Simulation Interaction Diagram tool, comprehensively interpreting outcomes for deviation, fluctuations, and intermolecular interactions [17]. Furthermore, the MM\GBSA studies were performed on the MD Simulation’s trajectories to compute the binding free energy and total complex energy. We have used the following commands in Ubuntu 22 for running the analysis:

export SCHRODINGER = /opt/Schrodinger2024-1
$SCHRODINGER/run thermal_mmgbsa.py desmond_md_job_FILE-NAME-out.cms
  which has produced the complexes after computations and trajectories-mmgbsa-prime-out.csv files which were further used for the analysis [17,18,29].

## 3. Results

### 3.1 Molecular interaction analysis

**Fig 2:**
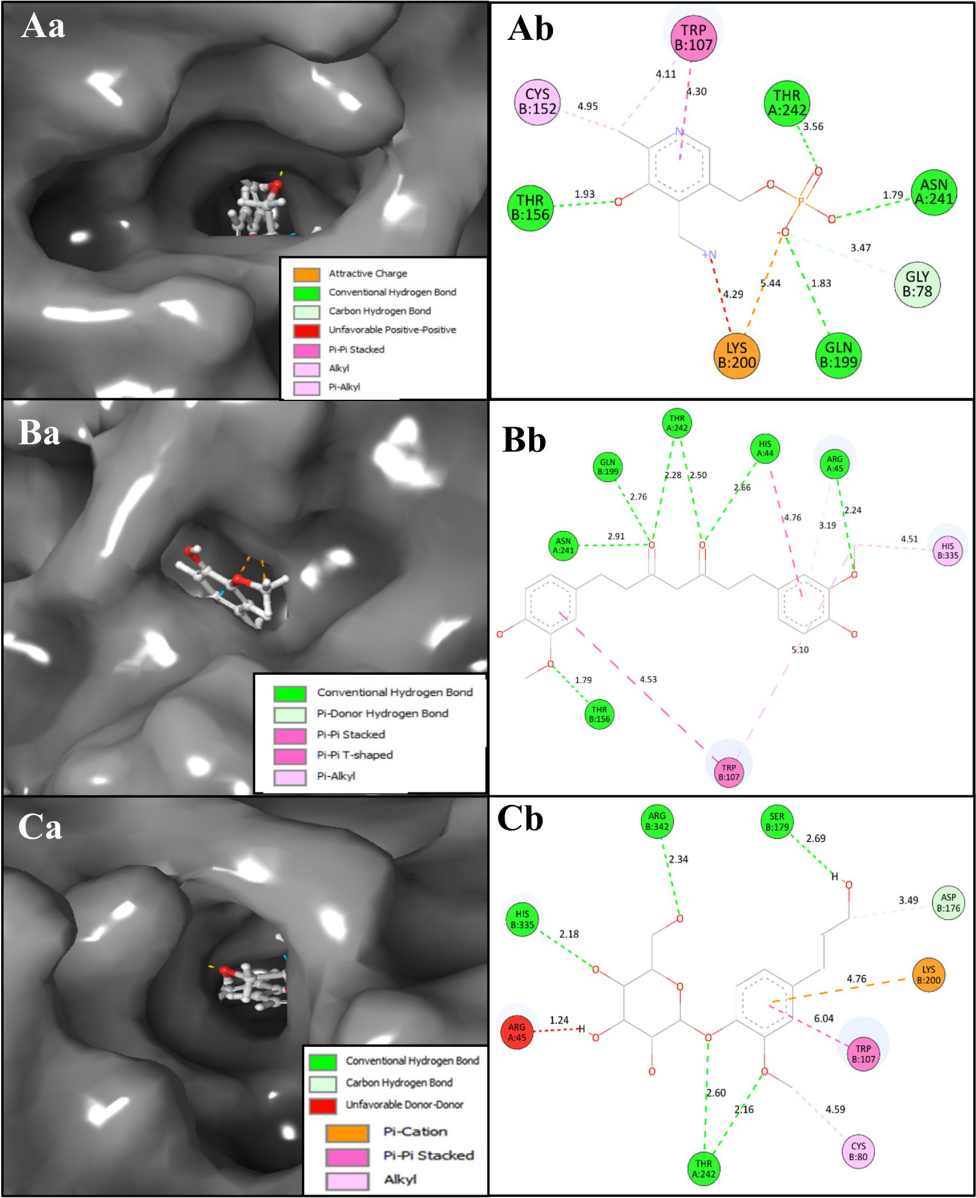
Showing the docked 3D and 2D docked poses of PSAT1 with Aa, Ab) Co-Crystallized ligand Pyridoxamine Phosphate, Ba, Bb) Tetrahydrocurcumin, and Ca, Cb) Coniferin. The legend is shown to understand the bond and residues’ types.

A molecular docking study of 417 molecules provided insight into potential inhibitors of PSAT1, 10 of the screened compounds had docking scores ≤ −8.5 kcal/mol (Table 1). Coniferin and Tetrahydrocurcumin were chosen as they are among the topmost (negatively) performed compounds, and their favourable ADME properties as they do not penetrate the BBB barrier and will be suitable natural candidates for ovarian cancer. These scores imply a high binding affinity and suggest that these compounds could be effective PSAT1 inhibitors, but ten molecules formed pi-pi bonds with Trp107. Trp107 plays an important function in the binding pocket of PSAT1, cofactor orientation, and catalysis of 3-PHP (Fig 2). Molecular docking revealed a strong interaction between PSAT1 and Coniferin (PubChem CID: 5280372) with a binding energy of -8.8 kcal/mol, six hydrogen bonds interact among HIS44, ARG45, SER179, and SER342 residues along 5 OH atoms, and THR242 residue interact along O atom. Further, a pi-pi stacking and pi-cation contact with TRP107 residue and LYS200 residue along the same benzene ring of the Coniferin ligand. Also, the Interaction between Tetrahydrocurcumin (PubChem CID: 124072) and PSAT1 showed a docking score of -8.9 kcal/mol (Table 1). Four hydrogen bonds interact among ASN154 residue with OH atom, GLY79, and THR242 residues with 2O atoms. Additionally, three pi-pi stacking contact HIE44 and TRP107 residues with two different benzene rings and a pi-cation contact LYS200 residue with the benzene ring of the Tetrahydrocurcumin ligand. This suggests a favourable binding affinity, indicating the potential of Coniferin as an inhibitor for PSAT1 (Fig 2). Coniferin and Tetrahydrocurcumin formed pi-pi bonds with Trp107, making it a potential inhibitor for PSAT1. These findings underscore the favourable binding affinities of Coniferin and Tetrahydrocurcumin as potential PSAT1 inhibitors (Fig 2). Notably, their interaction with Trp107, forming pi-pi bonds, further suggests their potential efficacy in inhibiting PSAT1 and warrants further experimental validation. Further, we also have shown the structures of the compounds in S1 Fig for better understanding.

**Table 1:** Molecular docking results against the Traditional Chinese Medicine and ADME analysis.

Ligand Name	PubChem CID	Docking score (kcal/mol)	Residues interacted. (H-bond, Pi-Alkyl, Pi-Sulphur, Pi-Sigma and Pi-Pi)	Swiss ADME
Pyridoxamine Phosphate (**Co-Crystallized ligand**)	-	-7.8	Trp107, Thr242, Asn241, Gln199, Thr156, Lys200, Cys152, Gly78	Co-Crystallized ligand
Tetrahydrocurcumin	124072	-8.9	Trp107, Asn241, Gln199, Thr242, His44, Arg45, Thr156, His335	Inhibit CYP2D6, CYP3A4, 1 Brenk alert
1-Hydroxy-2-methylanthraquinone	160817	-9.5	Trp107, Thr156, Thr242, Cys80	Inhibit CYP1A2, CYP2C19 & CYP3A4, BBB permeable
Dicinnamoylmethane	390472	-8.7	Trp107, Gly79, Gln199, Pro12, Cys152, Thr242	Inhibit CYP1A2, CYP2C19 & CYP3A4, 2 Brenk alerts, BBB permeable
Isoliquiritigenin	638278	-8.5	Trp107, Arg45, Ser178, Asp176	Inhibit CYP1A2, CYP2C9 & CYP3A4, 1 Brenk alert
Chlorogenic Acid	1794427	-8.5	Trp107, Asp176, Thr242, His44, Cys152, Arg45, Arg342	Violation of Ghose, Veber, Egan, and Muegge rule, 1 PAINS and 2 Brenk alerts
Coniferin	5280372	-8.8	Trp107, His335, Arg342, Ser179, Thr242, Asp176, Lys200, Cys80	1 Ghose Violation
Harman	5281404	-9.0	Trp107, Asp176, Cys152, Lys200	Inhibit CYP1A2 & CYP3A4, 1 Muegge Violation, BBB permeable
1-(4-Hydroxy-3-methoxyphenyl)-5-phenyl-1,4-pentadien-3-one	69501712	-9.0	Trp107, Thr242, Thr156, Cys80, Ser178	Inhibit CYP1A2, CYP2C19, CYP2C9 & CYP3A4, BBB permeable, 1 Brenk alert
1-(4-Hydroxy-3-methoxyphenyl)-5-phenyl-1,4-pentadien-3-one	69501712	-9.2	Trp107, Gln199, Thr156, His44	Inhibit CYP1A2, CYP2C19, CYP2C9 & CYP3A4, BBB permeable, 1 Brenk alert
1-(4-Hydroxy-3-methoxyphenyl)-5-phenyl-1,4-pentadien-3-one	69501712	-8.9	Gln199, Thr156, Ser179, Thr242, Ser178, Trp107	Inhibit CYP1A2, CYP2C19, CYP2C9 & CYP3A4, BBB permeable, 1 Brenk alert

### 3.2 Pharmacokinetics and interaction fingerprints studies

The QikProp descriptors and their standard values were computed for Coniferin and Tetrahydrocurcumin. The analysis reveals that both compounds generally adhere to the standard values for various descriptors. For instance, regarding chemical functionalities like #acid, #amide, #amidine, and #amine, Coniferin and Tetrahydrocurcumin fall within the accepted range. Similarly, binary descriptors such as #in34 and #in56, as well as numeric descriptors like #noncon, #nonHatm, #ringatoms, #rotor, #rtvFG, and #stars, all align with the specified standards for both compounds. Noteworthy variations arise in properties such as #metab, #NandO, accptHB, and ACxDN^.5/SA. Tetrahydrocurcumin, the newly identified lead molecule, exhibits a higher value in #metab and #NandO compared to Coniferin. Additionally, Coniferin shows a higher value in accptHB compared to Tetrahydrocurcumin. These differences may indicate potential distinctions in pharmacokinetic behaviour between the two compounds. In terms of molecular properties, both compounds meet the standard range for descriptors like mol MW, dipole, EA(eV), FISA, FOSA, glob, IP(eV), Jm, PercentHumanOralAbsorption, PISA, PSA, QPlogBB, QPlogHERG, QPlogKhsa, QPlogKp, QPlogPC16, QPlogPo/w, QPlogPoct, QPlogPw, QPlogS, QPPCaco, QPPMDCK, QPpolrz, and others (Table 2). This suggests that Coniferin and Tetrahydrocurcumin possess drug-like properties, but the observed differences in specific descriptors warrant further investigation and experimental validation to draw better conclusive insights into their pharmacokinetic profiles and suitability.

**Table 2:** Shows the pharmacokinetic properties of Coniferin and Tetrahydrocurcumin against QikProp standard values (Highlighting drug-like properties).

Descriptors	Standard	Coniferin	Tetrahydrocurcumin	Descriptors	Standard	Coniferin	Tetrahydrocurcumin
#acid	0–1	0	0	HumanOralAbsorption	-	3	2
#amide	0–1	0	0	IP(eV)	7.9–10.5	8.894	8.839
#amidine	0	0	0	Jm	-	0.4836	0.024465
#amine	0–1	0	0	**mol MW**	**130.0–725.0**	**342.345**	**372.417**
#in34	-	0	0	**%HumanOralAbsorption**	**>80% is high, <25% is poor**	**60.684**	**83.365**
#in56	-	12	12	PISA	0.0–450.0	95.513	235.379
#metab	1–8	6	9	**PSA**	**7.0–200.0**	**131.398**	**111.959**
#NandO	2–15	8	6	QPlogBB	−3.0–1.2	-2.016	-2.074
#noncon	-	5	0	QPlogHERG	concern below −5	-4.366	-5.751
#nonHatm	-	24	27	QPlogKhsa	−1.5–1.5	-0.912	0.037
#ringatoms	-	12	12	QPlogKp	−8.0 –−1.0	-3.981	-2.999
**e**	**0–15**	**11**	**12**	QPlogPC16	4.0–18.0	11.302	13.004
#rtvFG	0–2	1	2	**QPlogPo/w**	**−2.0–6.5**	**-0.39**	**2.864**
#stars	0–5	0	1	QPlogPoct	8.0–35.0	23.368	19.448
**accptHB**	**2.0–20.0**	**11.7**	**7**	QPlogPw	4.0–45.0	19.624	11.418
ACxDN^.5/SA	0.0–0.05	0.0445843	0.0144982	QPlogS	−6.5–0.5	-1.869	-4.184
Category	-	small	small	QPPCaco	<25 poor, >500 great	102.968	164.159
CIQPlogS	−6.5–0.5	-2.157	-4.694	QPPMDCK	<25 poor, >500 great	42.384	70.169
**CNS**	**−2 (inactive), +2 (active)**	**-2**	**-2**	QPpolrz	13.0–70.0	29.836	37.21
dip^2/V	0.0–0.13	0.012022	0.0430895	**RuleOfFive**	**maximum is 4**	**0**	**0**
dipole	1.0–12.5	3.558	7.25	**RuleOfThree**	**maximum is 3**	**0**	**1**
**donorHB**	**0.0–6.0**	**5**	**2**	SAamideO	0.0–35.0	0	0
EA(eV)	−0.9–1.7	0.329	0.132	SAfluorine	0.0–100.0	0	0
FISA	7.0–330.0	209.132	187.771	SASA	300.0–1000.0	586.798	682.807
FOSA	0.0–750.0	282.153	259.657	Volume	500.0–2000.0	1053.003	1219.921
glob	0.75–0.95	0.8530268	0.808639	WPSA	0.0–175.0	0	0

**Fig 3:**
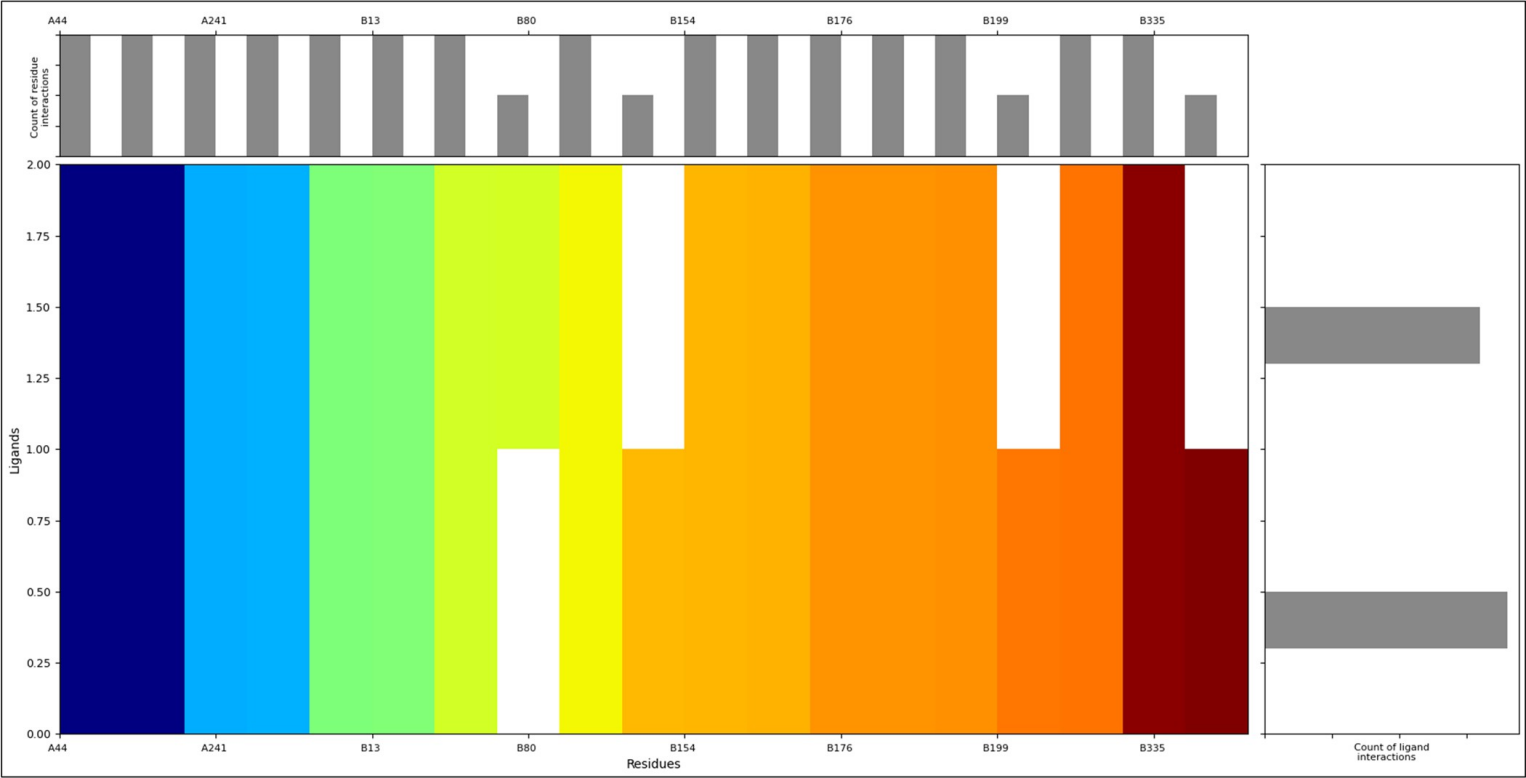
Showing the Molecular Interaction Fingerprints of docked poses, which shows the interacting residue and counts as follows- 3ARG, 4ASN, 2ASP, 2CYS, 2GLN, 4GLY, 4HIE, 2LYS, 2PRO, 4SER, 4THR, and 2TRP (upper bar graph). Coniferin has shown more interactions than Tetrahydrocurcumin (right bar graph), and the N to C terminals of the proteins are coloured and shown from blue to red to make the interaction pattern clear.

The computed molecular interaction fingerprints highlight diverse amino acid residues and their respective counts, shedding light on the potential molecular interactions within the analysed compounds. Among these, three Arginine (ARG) residues indicate involvement in interactions with positively charged functional groups, while the presence of four Asparagine (ASN) residues suggests participation in interactions related to polar functional groups. Being negatively charged, two Aspartic Acid (ASP) residues may contribute to interactions with negatively charged groups. Cysteine (CYS), with two instances, may be involved in interactions related to sulfur-containing functional groups, potentially forming disulfide bonds. Two Glutamine (GLN) residues, being polar and uncharged, could participate in interactions with polar functional groups. Four Glycine (GLY) residues suggest flexibility in molecular structures, facilitating interactions. Four Histidine (HIE) residues, known for their versatility, could be engaged in various interaction types. Two Lysine (LYS) residues, being positively charged, may interact with negatively charged groups. Two Proline (PRO) residues, known for introducing rigidity, could influence the conformation of molecular interactions. Four Serine (SER) and four Threonine (THR) residues, both polar and uncharged, may contribute to interactions with polar functional groups. With their aromatic nature, two Tryptophan (TRP) residues might be involved in hydrophobic interactions (Fig 3). This detailed description of the identified residues and their counts in Fig 3 provides valuable insights into the potential diversity and complexity of molecular interactions within the analysed compounds.

### 3.3 Molecular dynamics simulation studies

Molecular Dynamics (MD) simulation is a vital technology that is becoming increasingly popular in drug discovery and provides a deep understanding of the complex dynamics, functional features, and behaviour of drug-target complexes. The 100 ns production run examined the detailed deviations, fluctuations, and intramolecular interaction patterns that appeared during the simulation. Further, the detailed details are as follows.

#### 3.3.1 Root-mean-square deviation

**Fig 4:**
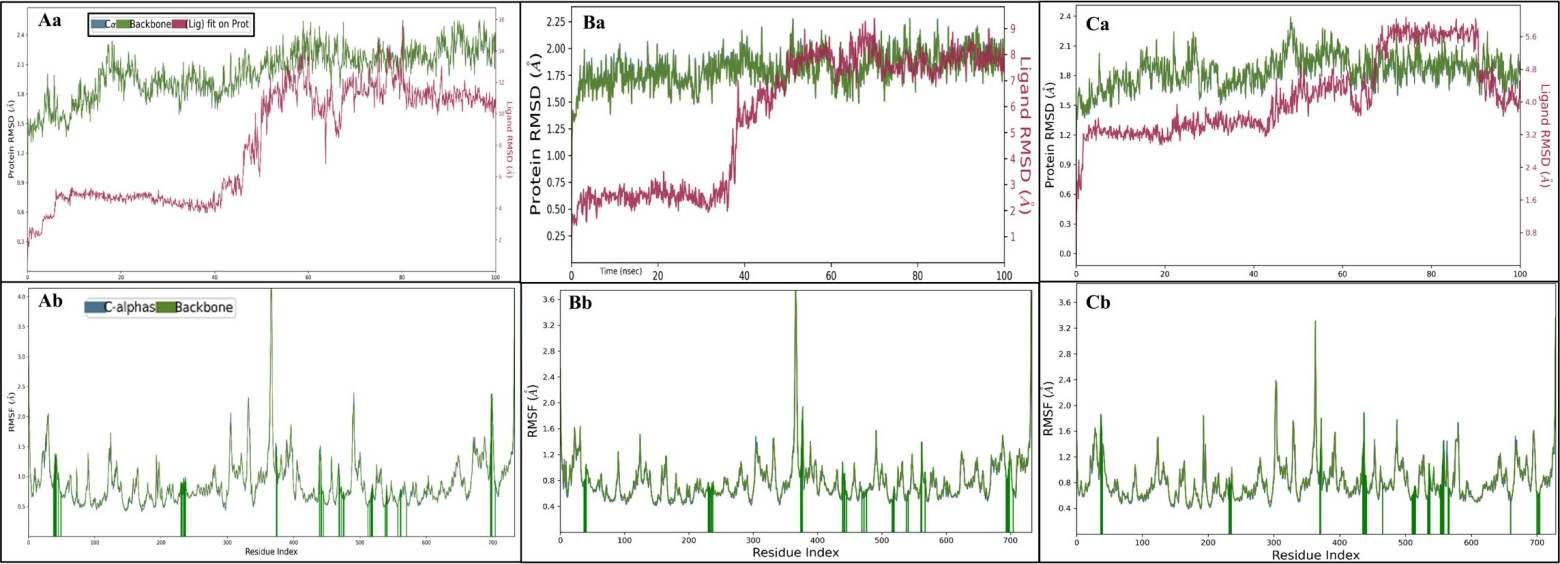
Showing the Root Mean Square Deviation of PSAT1 in complex with **Aa)** Coniferin, **Ba)** Tetrahydrocurcumin Ca) PMP where red indicates the deviation in ligand, blue shows the Cα, and the light green shows the protein’s backbone. The Root Mean Square Fluctuations of PSAT1 are shown where the dark green line shows the interactions of **Ab)** Coniferin, **Bb)** Tetrahydrocurcumin and Cb) PMP with PSAT1.

Root mean square deviations (RMSD) are used to evaluate the deviations in ligands and proteins at the nanosecond level of simulation. This approach provides a comprehensive analysis during simulation by enabling a molecular-scale level of compound complexity and stability. In case of PSAT1 complex interacting with co-crystallized ligand (PMP), initial deviations observed at 0.10 ns 1.48 Å for protein and 1.30 Å for ligand. Upon stabilization of compound at 100 ns the deviation for protein was observed at 1.81 Å and for ligand, the deviation was observed at 4.51 Å. Furthermore, initial deviations were observed in the study of the PSAT1 complex interacting with Coniferin; these variations indicated deviations of 1.55 Å for the protein and 2.11 Å for the ligand at 0.10 ns. Still, as the simulation went on, the complex stabilised. The ligand deviation significantly extended to 10.93 Å, whereas the protein deviation only marginally increased to 2.41 Å at 100 ns. However, general stability was maintained, except for the first one-ns interval (Fig 4Aa). Similarly, in the complex of PSAT1 with Tetrahydrocurcumin (PubChem ID: 124072), early deviations were followed by a stabilising tendency. The protein and ligand showed variations of 1.04 Å and 1.47 Å, respectively, at 0.10 ns. During the simulation period, at 100 ns, the ligand deviation marginally increased to 7.64 Å, while the protein deviation increased to 1.89 Å (Fig 4Ba). Computational simulations assessed the complexity and robustness of PSAT1 complexes containing Coniferin and Tetrahydrocurcumin. While some early deviations were observed in most cases, these complexes were consistently stable and performed well throughout the experiment. Differences in the protein and ligand structures provide important molecular-level information that makes a thorough evaluation of the compound’s activity possible.

#### 3.3.2 Root-mean-square fluctuation

Root Mean Square Fluctuation (RMSF) quantifies the average fluctuations of atomic positions within a molecular structure over time, indicating flexibility. It is vital in analysing protein dynamics, revealing regions with high fluctuations. In the PSAT1 in complex with PMP residues that deviated beyond 2 Å include ALA310, LYS311, LEU370, and LEU370. Whereas the residues interacting with PMP involve SER43, HIS44, ARG45, LEU239, TYR240, ASN241, PRO12, GLY13, GLY77, GLY79, GLN82, PHE83, TRP107, TYR151, ASN154, GLU155, THR156, VAL157, ASP176, SER178, SER179, PHE195, GLY197, GLN199, LYS200, VAL207, ASN302, ARG342, SER344, and TYR346. In case of PSAT1 complex with Coniferin, residues that deviate beyond 2Å include GLN6, VAL35, ALA310, ARG336, SER337, LEU370, LEU128, GLY129, ARG336, SER337, and VAL338. These residues play a crucial role in the structural dynamics of the complex. Conversely, several residues of PSAT1 interact with Coniferin, contributing to the stability of the complex. These interacting residues involve SER43, HIS44, ARG45, SER46, SER47, ALA50, ASN54, GLY235, ASN236, SER237, LEU239, TYR240, ASN241, THR242, PRO12, GLY13, GLY77, GLY79, CYS80, PHE83, ALA106, TRP107, LYS110, GLU113, GLU114, TYR150, CYS152, ASN154, GLU155, THR156, VAL157, ASP176, SER178, SER179, PHE195, GLN199, LYS200, THR208, HIS335, ARG336, SER337, and ARG342 (Fig 4Ab). These interactions contribute to the overall stability and functionality of the PSAT1-Coniferin complex. Similarly, in the case of PSAT1 in complex with Tetrahydrocurcumin, residues deviating beyond 2Å include.LEU370, GLN6, and LEU370. These deviations highlight the structural changes induced by Tetrahydrocurcumin binding. The interaction between PSAT1 and Tetrahydrocurcumin involves residues such as MET42, SER43, HIS44, ARG45, GLY235, ASN236, SER237, LEU239, TYR240, THR242, PRO12, GLY13, PRO14, GLY77, GLY79, CYS80, PHE83, TRP107, LYS110, GLU114,ASN154, GLU155, THR156, VAL157, ASP176, SER179, GLN199, LYS200, ALA205, SER331, LYS333, GLY334, HIS335,ARG336, ARG342. These interactions contribute to the stability and conformational changes in the PSAT1-Tetrahydrocurcumin complex. This detailed analysis clarifies the specific residues involved in the deviations and interactions within the PSAT1 complexes with Coniferin and Tetrahydrocurcumin (Fig 4Bb).

#### 3.3.3 Simulation interaction analysis

**Fig 5:**
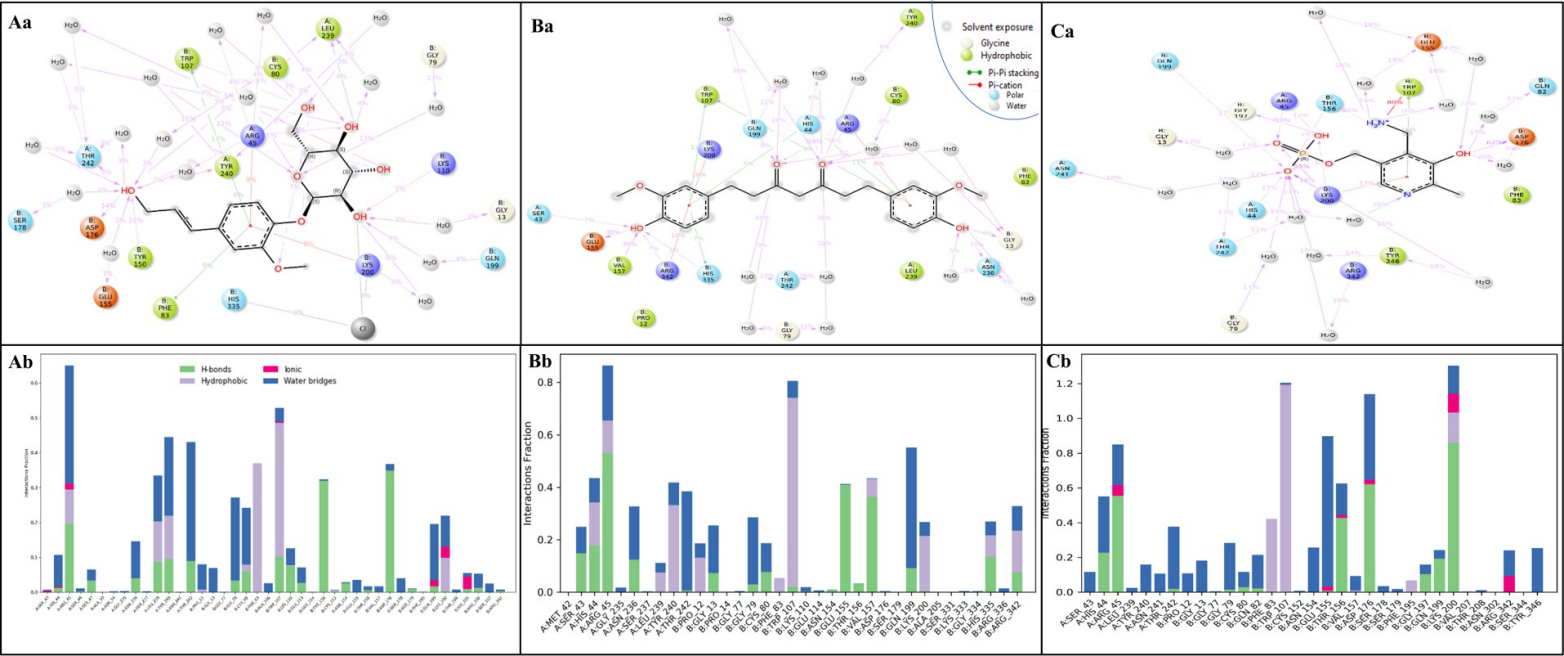
Showing the Simulation Interaction Diagram of PSAT1 in complex with **Aa)** Coniferin, **Ba)** Tetrahydrocurcumin and Ca) PMP and the count of interactions of are shown for **Ab)** Coniferin, **Bb)** Tetrahydrocurcumin and Cb) PMP where red shows the ionic, blue shows the water bridges, grey shows the hydrophobic interaction while the light green shows the H-bonds.

The Simulative Interaction Diagram visually shows the interaction between the protein and ligand during the simulation. The different angles, configurations, and ideal binding poses that arise during the interaction between the ligand and protein are dynamically depicted in this diagram. The complex with PSAT1 and Coniferin involves hydrogen bonds among GLY79 and TYR240 residues with water molecules along the O atom, TYR150, and ASP176 residue along the OH atom, and to neutralize the complex added Cl ion along the OH atom with metal coordination. Additionally, two pi-pi stackings contact PHE83 and TRP107 residues, and two pi-cations contact ARG45 and LYS200 residues with the same benzene ring of the Coniferin ligand (Fig 5Aa) and Fig 5Ab shows the count of interaction in the histogram. In the complex with PSAT1 and Tetrahydrocurcumin (PubChem ID: 12402), many hydrogen bonds interacted among GLU155, VAL157, and ASN236 residue 2 OH atoms, and GLY79, GLN199, THR242 residues with water molecules and HIS44, ARG45 residue along 2 O atoms. However, two pi-pi stackings and 2 pi-cation contact TRP107, HIS44 residue, and LYS200, ARG342 residue with 2 benzene rings of the Tetrahydrocurcumin ligand (Fig 5Ba), and the count of interactions are shown in Fig 5Bb.

### 3.4 Molecular mechanics, general born surface area (MM\GBSA) studies

MM\GBSA studies on MD (Molecular Dynamics) simulation trajectory files provide critical insights into binding free energies and stability of ligand-receptor complexes. By analyzing snapshots from MD simulations, MM\GBSA calculates the binding affinities and energetics, offering a detailed understanding of the interactions at the atomic level. This method is essential for drug discovery, optimizing lead compounds, and understanding molecular mechanisms in biological systems. Table 3 presents a comparative analysis of Coniferin, Tetrahydrocurcumin and PMP based on 1001structures each. Tetrahydrocurcumin shows an average binding free energy (dG) of -52.4347 kcal/mol with a standard deviation of 7.28, ranging from -74.5241 to -31.7729 kcal/mol. The average non-solvated binding energy (dG(NS)) is -62.6535 kcal/mol with a standard deviation of 6.46, ranging from -82.6793 to -40.9262 kcal/mol. In comparison, Coniferin has an average dG of -41.3451 kcal/mol with a standard deviation of 9.65, ranging from -79.6010 to -17.7366 kcal/mol, and an average dG(NS) of -44.2986 kcal/mol with a standard deviation of 9.96, ranging from -86.9247 to -20.4439 kcal/mol. Whereas PMP has an average dG of -32.1660 kcal/mol with a standard deviation of 3.25, ranging from -52.3986 to -21.7583 kcal/mol, and an average dG(NS) of -34.4258 kcal/mol with a standard deviation of 3.29, ranging from -55.0124 to -23.9652 kcal/mol. All ligands have processed 1001 frames, resulting in 1001 output structures.

**Table 3:** Showing various energies computed during the MM\GBSA computations.

Metric	Tetrahydrocurcumin	Coniferin	PMP
Number of structures	1001	1001	1001
dG Average (kcal/mol)	-52.4347	-41.3451	-32.1660
dG Standard Deviation	7.28	9.65	3.25
dG Range (kcal/mol)	-74.5241 to -31.7729	-79.6010 to -17.7366	-52.3986 to -21.7583
dG(NS) Average (kcal/mol)	-62.6535	-44.2986	-34.4258
dG(NS) Standard Deviation	6.46	9.96	3.29
dG(NS) Range (kcal/mol)	-82.6793 to -40.9262	-86.9247 to -20.4439	-55.0124 to -23.9652
Number of frames processed	1001	1001	1001
Number of output structures	1001	1001	1001

**Fig 6:**
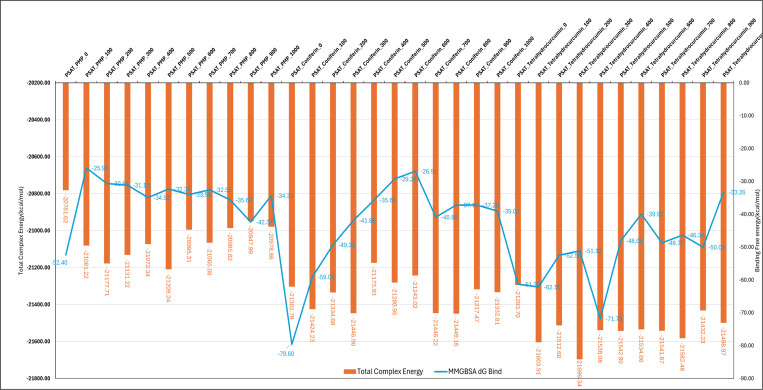
Showing the binding free energy and total complex energy of the PSAT1 complex with Coniferin and Tetrahydrocurcumin.

The analysis of Coniferin and Tetrahydrocurcumin complexes based on their MM\GBSA binding free energy (dG Bind) and total complex energy across different frames reveals significant insights into their stability. In case of PMP, it shows an overall poor MM\GBSA dG Bind ranging from -52.3986 to -21.7583 from 0 to 1000 frames indicating minor fluctuations with a standard deviation of 3.29. For Coniferin, the MM\GBSA dG Bind ranges from -79.60 kcal/mol at frame 0 to -26.91 kcal/mol at frame 600, indicating fluctuations in binding affinity over time. The total complex energy for Coniferin varies from -21449.16 kcal/mol at frame 800 to -21173.93 kcal/mol at frame 400, suggesting some variability in the overall stability of the complex. In contrast, Tetrahydrocurcumin shows a dG Bind range from -71.79 kcal/mol at frame 400 to -33.26 kcal/mol at frame 1000, demonstrating similar fluctuations in binding affinity. The total complex energy for Tetrahydrocurcumin ranges from -21696.34 kcal/mol at frame 300 to -21293.70 kcal/mol at frame 0, indicating a broader range of stability compared to Coniferin. The average binding free energy (dG Bind) values indicate that Tetrahydrocurcumin generally has a stronger binding affinity (-52.4347 kcal/mol) compared to Coniferin (-41.3451 kcal/mol) (Fig 6). The lower standard deviation in Tetrahydrocurcumin’s dG Bind (7.28 kcal/mol) compared to Coniferin (9.65 kcal/mol) suggests that Tetrahydrocurcumin’s binding interactions are more consistent over time. The non-solvated binding energy (dG(NS)) for Tetrahydrocurcumin is lower (-62.6535 kcal/mol) compared to Coniferin (-44.2986 kcal/mol), further supporting the stronger binding affinity of Tetrahydrocurcumin. MM\GBSA analysis indicates that Tetrahydrocurcumin forms a more stable complex with a higher and more consistent binding affinity than Confierin (Fig 6). The variations in the total complex energy and binding free energy across frames reflect the dynamic nature of molecular interactions and the influence of different conformations sampled during the molecular dynamics simulation. Despite the fluctuations, Tetrahydrocurcumin maintains a more stable and stronger binding profile than Confierin and PMP, which is crucial for drug design and optimization processes.

## 4 Discussion

Ovarian cancer is a formidable gynaecological malignancy with a high mortality rate and late-stage diagnosis, posing a significant global health challenge. The study aims to explore a novel approach for early-stage ovarian cancer treatment by computationally identifying and validating potential Traditional Chinese Medicine (TCM) inhibitors targeting PSAT1, which is a crucial enzyme in the serine biosynthesis pathway, essential for the growth and survival of cancer cells, particularly in the early stages of ovarian cancer. The significance of this study lies in its innovative approach to leveraging computational methods for drug discovery. Traditional Chinese medicine, with its holistic approach and abundant natural substances, is explored for its potential to inhibit PSAT1 in early-stage ovarian cancer. The study addresses the limitations of current treatments and aims to contribute to developing new therapeutic approaches. The methodology involves a systematic approach, starting with collecting and preparing protein and ligand data. The PSAT1 structure is obtained from RCSB PDB, and a TCM library of 238 compounds is selected for screening. Molecular docking is performed using AutoDock Vina to predict the preferred orientation of TCM compounds binding to PSAT1. The pharmacokinetics and molecular interaction fingerprints of the identified compounds, Coniferin and Tetrahydrocurcumin, are evaluated using the QikProp tool in Maestro. A 100ns MD simulation is also conducted to study the stability, deviations, and intermolecular interactions. The molecular docking results reveal that Coniferin and Tetrahydrocurcumin exhibit high binding affinities with PSAT1, suggesting their potential as inhibitors. The pharmacokinetics studies show that both compounds generally adhere to standard values for various descriptors, indicating their drug-like properties. Molecular interaction fingerprints highlight diverse amino acid residues involved in interactions, providing valuable insights into the potential molecular interactions within the compounds. MD simulation studies demonstrate the stability and performance of the PSAT1 complexes with Coniferin and Tetrahydrocurcumin over the 100ns simulation period. Coniferin and Tetrahydrocurcumin emerge as potential inhibitors with high docking scores and significant interactions with critical residues, including Trp107, essential for PSAT1’s function. The pi-pi stacking and pi-cation contacts further support their potential efficacy. The QikProp analysis indicates that Coniferin and Tetrahydrocurcumin possess drug-like properties, but some differences in specific descriptors suggest potential distinctions in pharmacokinetic behaviour. The interaction fingerprints reveal the diversity and complexity of molecular interactions within the compounds, emphasising their potential in modulating PSAT1. The MD simulation results show that Coniferin and Tetrahydrocurcumin complexes with PSAT1 exhibit stability, with initial deviations followed by a stabilising tendency. The RMSD and RMSF analyses provide insights into the dynamic behaviour of the complexes, highlighting specific residues involved in deviations and interactions. The docking results and the residues that participated during the interactions were also found in the simulation interaction diagram making the dynamics results more reliable with the validation step and the energy computed during the MM\GBSA again makes it more towards getting the results validated. Tetrahydrocurcumin is a metabolite of curcumin, the active ingredient in turmeric. It has been shown to have several anticancer properties, including inhibiting tumour growth, inducing apoptosis, and reducing inflammation [31,32]. Coniferin is a glycoside in many plants, including conifers and pine trees. It has also been shown to have anticancer properties, including inhibiting tumour growth and inducing apoptosis [33]. The comprehensive MM\GBSA studies show the variations in total complex energy and binding free energy across different frames, highlighting the dynamic nature of molecular interactions and the impact of various conformations sampled during the molecular dynamics simulation. Despite these fluctuations, Tetrahydrocurcumin consistently demonstrates a more stable and stronger binding profile compared to Coniferin, which is essential for drug design and optimization efforts.

## 5 Conclusions

The computational studies support the hypothesis that Coniferin and Tetrahydrocurcumin can be potent inhibitors of PSAT1 in early-stage ovarian cancer, and both are natural compounds, relatively non-toxic, making them good candidates for further development as PSAT1 inhibitors. The results from molecular docking, pharmacokinetics studies, interaction fingerprints, MD simulations, and the additional MM\GBSA computations collectively provide a comprehensive understanding of the potential of these TCM compounds. However, experimental studies are crucial to validate these findings and confirm the inhibitory effects of Coniferin and Tetrahydrocurcumin on PSAT1. This study opens the door for further preclinical and clinical research, emphasising the potential of computational approaches in drug discovery for ovarian cancer treatment and we also encourage the inclusion of other drug libraries for more efficient drug designing for early and proper ovarian cancer therapeutics.

## Supporting information

S1 Fig Structure of the top 10 screened compounds. (TIF)
